# Knowledge and perceptions of cardiopulmonary resuscitation amongst New Zealand podiatrists: a web-based survey

**DOI:** 10.1186/s13047-021-00481-9

**Published:** 2021-05-14

**Authors:** Angela Brenton-Rule, Daniel Harvey, Kevin Moran, Daniel O’Brien, Jonathon Webber

**Affiliations:** 1 School of Clinical Sciences, Faculty of Health and Environmental Sciences, Auckland University of Technology, Private Bag 92006, 1142 Auckland, New Zealand; 2 Sports & Spinal Physiotherapy, Westgate, Auckland, New Zealand; 3 Faculty of Education, The University of Auckland, Auckland, New Zealand; 4 Department of Anaesthesiology, School of Medicine, Faculty of Medical and Health Sciences, The University of Auckland, Private Bag 92019, 1142 Auckland, New Zealand

**Keywords:** New Zealand, podiatry, cardiopulmonary resuscitation, knowledge and beliefs

## Abstract

**Background:**

Podiatrists in New Zealand have a duty of care to assist patients in an emergency, and current cardiopulmonary resuscitation (CPR) certification is a requirement for registration. However, it is unknown how competent and confident podiatrists are in administering CPR and how they would respond in an emergency. Having a health professional who has a competent knowledge of CPR and skills in basic life support, can improve survival rates from sudden cardiac arrest. Therefore, the aim of this study was to survey New Zealand podiatrists to determine their CPR knowledge and qualifications; beliefs about the application of CPR; and perceptions of their competency in CPR.

**Methods:**

This cross-sectional study used a web-based survey. Participants were New Zealand registered podiatrists with a current annual practising certificate. The 31-item survey included questions to elicit demographic information, CPR practice and attitudes, and CPR knowledge. Responses were collected between March and August 2020.

**Results:**

171 podiatrists responded to the survey. 16 % of the podiatrists (*n* = 28) had performed CPR in an emergency, with a 50 % success rate. Participants were predominantly female (*n* = 127, 74 %) and working in private practice (*n* = 140,82 %). Nearly half of respondents were younger than 40 years (*n* = 75,44 %) and had less than 10 years of clinical experience (*n* = 73, 43 %). Nearly all (*n* = 169,97 %) participants had received formal CPR training in the past two years, with 60 % (*n* = 105) receiving training in the past 12 months. Most respondents (*n* = 167,98 %) self-estimated their CPR ability as being effective, very effective, or extremely effective. Participants’ knowledge of CPR was variable, with the percentage of correct answers for CPR protocol statements ranging between 20 and 90 %.

**Conclusions:**

This study provides the first insight into New Zealand podiatrists’ CPR knowledge and perceptions. Podiatrists were found to have high levels of CPR confidence but demonstrated gaps in CPR knowledge. Currently, New Zealand registered podiatrists require biennial CPR re-certification. However, resuscitation authorities in New Zealand and overseas recommend an annual update of CPR skills. Based on this study’s findings, and in line with Australia and the United Kingdom, the authors recommend a change from biennial to annual CPR re-certification for podiatrists in New Zealand.

**Trial registration:**

The study was registered with the Australian New Zealand Clinical Trials Registry (ACTRN12620001144909).

**Supplementary Information:**

The online version contains supplementary material available at 10.1186/s13047-021-00481-9.

## Background

Cardiopulmonary resuscitation (CPR) is an essential life-saving skill when applied to people who are unresponsive and not breathing due to cardiac arrest. CPR involves a combination of chest compressions and rescue breathing (ventilation), in a 30:2 ratio, according to best practice guidelines [1]. CPR aims to maintain a circulation sufficient to preserve brain function until specialised equipment, such as an Automated External Defibrillator (AED), becomes available. Early, high-quality CPR has been shown to save lives and improve patients’ neurological outcomes [1–5].

In New Zealand, nearly 2,000 people per year (approximately five per day) are attended by St John and Wellington Free Ambulance Services for an out-of-hospital cardiac arrest, with 76 % of patients receiving bystander CPR [6]. To improve the rates of bystander CPR and AED use, the New Zealand Resuscitation Council and its member organisations promote and support community initiatives to increase the availability of CPR and AED training to the public [7]. However, there is no legal obligation for a member of the public to act in a medical emergency.

In contrast, healthcare professionals have a duty of care and legal obligations under the New Zealand Crimes Act 1961 (s151) and the Health and Disability Commissioner Act 1994 (Right 4s2 of the Code of Health and Disability Services Consumers’ Rights) to attend to a medical emergency for people in their care [8]. Healthcare professionals who have competent knowledge of CPR and skills in basic life support, can improve the survival rates of people who experience cardiac arrest [8]. As such, the Australian and New Zealand Committee on Resuscitation (ANZCOR) additionally recommends that healthcare professionals (either on or off-duty) should assist in an emergency, if requested to do so [8].

Podiatrists are registered healthcare professionals requiring an annual practising certificate, regulated and administered by the Podiatrists Board of New Zealand (PBNZ). In addition to their obligations under the law, podiatrists must adhere to the Principles and Standards for the Practice of Podiatry in New Zealand [9]. This includes the principle of safe practice which requires podiatrists to keep professional knowledge and skills up to date through continuing medical education. The continuing medical education requirements to maintain an annual practising certificate includes current (within two years) CPR certification and anaphylaxis management. However, whilst this is a mandatory requirement, practitioner competence and confidence to perform CPR in an emergency are not monitored by the PBNZ.

There are approximately 450 registered podiatrists in New Zealand, with the majority being self-employed [10]. Podiatrists treat patients with diverse health backgrounds and in various settings ranging from private practice, hospitals, residential aged care and the community, including private homes, Marae (the traditional meeting place of indigenous New Zealanders), and sporting venues [10]. In these settings, podiatrists may be working in multi-disciplinary teams, in isolation from medical personnel or be the only recognised healthcare professional providing care.

Like many countries, New Zealand is experiencing a rapid and substantial increase in people aged over 65 [11]. The older population is also becoming more ethnically diverse with an increase in Māori (the indigenous people of New Zealand) and Pasifika (people who originate from islands of the South Pacific region). Māori and Pasifika typically experience poorer health outcomes, compared to non-Māori, and have a higher incidence of out-of-hospital cardiac arrest with lower survival rates [12]. One in six older people live with three or more long term conditions, such as diabetes and heart disease [11], and these figures are higher in some ethnic groups within New Zealand [12]. Foot problems associated with ageing, cardiovascular disease and diabetes are common, and a significant part of podiatry practise [13, 14]. New evidence is also emerging on the impact of COVID-19 on the feet and cardiorespiratory system [15, 16]. Therefore, over time, the increase in people with chronic conditions, presenting to podiatry, may expose podiatrists to an increasing number of medical emergencies requiring CPR intervention.

Previous research has investigated the attitude, knowledge, and practice of CPR amongst doctors, nurses, lifeguards, paramedics, dentists, radiographers, physiotherapists, and dental and medical students [17–28]. Furthermore, international research into health professionals’ attitudes, beliefs, and knowledge of CPR has led to changes in training and improvement in CPR skills and practice [25, 29, 30]. However, whilst podiatrists in New Zealand are required legally to maintain current CPR certification and have a legal and ethical duty to assist in a medical emergency, the competence and confidence of New Zealand podiatrists in administering CPR is unknown.

To date, the authors are unaware of research globally investigating the attitude, knowledge, and practice of CPR amongst podiatrists. Therefore, this study aimed to survey New Zealand podiatrists to determine their CPR knowledge and qualifications, beliefs about CPR use in an emergency, and perceptions of CPR competency.

## Methods

The research design was a cross-sectional observational study using a web-based survey. The survey was advertised on the NZ Podiatry Alumni Facebook page and through email invitation via the PBNZ. Participants comprised a convenience sample of New Zealand registered podiatrists who were practising at the time of data collection. Ethical approval was obtained from the Auckland University of Technology Ethics Committee (Reference number 20/30) before data collection commenced. The study was registered with the Australian New Zealand Clinical Trials Registry (ACTRN12620001144909).

The survey was developed collaboratively by the authors and pilot-tested by a small group of podiatrists. Survey questions were initially developed from a previous study of New Zealand physiotherapists [28], and revisions were made based on feedback from pilot testing. The survey comprised 31 closed questions divided into three sections. The first section focussed on demographics (questions 1–6) and sought information on gender, age, years of podiatry experience, postgraduate qualification, employment status and work setting. The second section focussed on CPR practice and attitudes (questions 7–20), including: questions on most recent formal CPR training; whether the training included anaphylaxis management and AED use; if training included face-to-face teaching; current CPR certification; knowledge of the location of the nearest AED; previous application of CPR and outcomes; and estimated success rate of CPR in out-of-hospital cardiac arrest. Six questions using Likert-type scales asked respondents about their perceptions of CPR. A five-point scale ranging from poor to highly effective was used to assess their self-estimated ability to perform CPR. Other questions used a five-point scale ranging from strongly disagree to strongly agree about confidence to use CPR at work; sense of duty to perform CPR at work; confidence to use CPR in the public domain; the necessity for self-protection before performing CPR; and mouth to mouth ventilation. The final section focused on CPR knowledge (questions 21–31) and used true/false responses to 11 statements on CPR protocols from the New Zealand Resuscitation Council [1]. The survey was hosted on the internet-based survey site SurveyMonkey® which enabled anonymous, self-administered participation. Consent was obtained via submission of the completed survey. The survey was open between March and August 2020. An adapted version of the online survey is presented in Additional File 1.

Data from the completed surveys were entered in IBM SPSS Statistics for Windows, Version 26 for statistical analysis. Descriptive statistics characterised all numeric variables using frequency and percentages. Chi-square statistics of independence were used to test associations between the independent sociodemographic variables of: age; sex; years of podiatry experience; employment status; place of work; and postgraduate qualification, against CPR training; beliefs; and knowledge.

## Results

38 % (*n* = 174/453) of all New Zealand registered podiatrists with an annual practising certificate participated in the study. Three individuals who enrolled in the survey were excluded because they did not answer the questions relating to CPR, leaving a sample size of 171 registered podiatrists.

### Podiatrist demographics

The sample included more females than male podiatrists (female 74 %, male 26 %). Almost half were aged less than 40 years (44 %) and had less than 10 years clinical experience (43 %) (Table 1). Most (82 %) worked in private practice and were either employed (37 %) or self-employed (57 %). These demographics are consistent with 2019 national data obtained by Carroll and colleagues [31], which reported that the New Zealand podiatry profession is mainly female (67 %), work in private practice (80 %), and are self-employed business owners (69 %) or employees (37 %).

**Table 1:** Characteristics of sample population (*N* = 171)

	*n*	*%*	*Cumulative %*
Gender			
Female Male	127 44	74.3 25.7	74.3 100.0
Age			
20–29 years	40	23.4	23.4
30–39 years	35	20.5	43.9
40–49 years	36	21.1	64.9
50–59 years	45	26.3	91.2
Over 60 years	15	8.8	100.0
Years of podiatry experience			
< 10 years	73	42.7	42.7
> 10 years	98	57.3	100.0
Postgraduate qualification			
Yes	52	30.4	30.4
No	119	69.6	100.0
Current employment status			
Employed	63	36.8	36.8
Self-employed	97	56.7	93.6
Employer	6	3.5	97.1
Other	5	2.9	100.0
Main work setting			
Private practice	140	81.9	81.9
Hospital/Clinic	15	8.8	90.6
Community	7	4.1	94.7
University	7	4.1	98.8
Other (sports team)	2	1.2	100.0

### CPR training and perceptions of its value

Most respondents (60 %) reported having received formal CPR training in the previous year. Almost all (97 %) had received training in the past two years (Table 2). Most instruction had included AED use (99 %), anaphylaxis management (87 %), and consisted of face-to-face training (98 %). Most respondents (88 %) knew the location of the nearest AED in relation to their workplace. Some respondents (16 %) had used CPR in an emergency and of these, one half (50 %) of the patients had been successfully resuscitated. Half of the podiatrists (53 %) accurately suggested a success rate of ≤ 25 % for CPR in out-of-hospital cardiac arrest.

**Table 2:** Cardiopulmonary resuscitation training and perceptions of its value (Q7-14)

		*n*	*%*	*Cumulative %*
Last formal CPR training	< 3 months	20	11.5	11.5
	3–12 months	85	48.9	60.4
	1-2 years	64	36.8	97.2
	>2 years	2	1.1	98.3
Included AED training	Yes	170	99.4	99.4
	No	1	0.6	100.0
Included anaphylaxis management	Yes	149	87.1	87.1
	No	22	12.6	100.0
Included face-to-face teaching	Yes	167	97.7	97.7
	No	4	2.3	100.0
Know where the nearest AED is relative to your workplace	Yes No	150 21	87.7 12.3	87.7 100.0
Have used CPR in an emergency	Yes No	28 143	16.4 83.6	16.4 100.0
If yes, was it successful?	Yes	14	50.0	50.0
	No	10	35.7	85.7
	Don’t know	4	14.3	100.0
What is the survival rate of out-of-hospital cardiac arrest?	0–25 %	92	52.9	52.9
	26–50 %	51	29.3	82.2
	51–75 %	23	13.2	95.4
	76–100 % Nil response	5 3	2.9 1.7	98.3 100.0

No significant differences were found in current CPR training or the topics taught when analysed by age, sex, years of experience, employment status, place of work, or postgraduate qualification. When asked whether participants had used CPR in an emergency, no significant differences were found when the data were analysed by age, sex, years of experience, employment status, place of work, or postgraduate qualification.

No significant differences were found when the estimated success rates of CPR in out-of-hospital cardiac arrests were analysed by sex, years of experience, work setting, employment status or postgraduate qualification. Significant differences were evident when out-of-hospital cardiac arrest estimates were analysed by age and years of experience. More participants aged 40 + years reported the correct survival rate (< 25 %), of people experiencing out-of-hospital cardiac arrest, compared with the younger participants (40 + years, 75 %; <40 years, 25 %) (χ2 (3) = 29.227, *p* = < 0.001). Similarly, more experienced podiatrists reported lower survival rates (< 25 %), of people experiencing out-of-hospital cardiac arrest, compared with the less experienced podiatrists (> 10 + years, 74 %; <10 years, 26 %) (χ2 (3) = 22.881, *p* = < 0.001).

### Beliefs about CPR use

Most respondents self-estimated their CPR competency as being effective (98 %). Of these, less than half (41 %) of the participants believed that their capacity to perform CPR was very effective or extremely effective (Table 3). No significant differences were found when this self-estimated ability was analysed by age, sex, experience, work status, work setting, or postgraduate qualification.

**Table 3:** Beliefs about cardiopulmonary resuscitation use

		*n*	*%*	*Cumulative %*
How would you rate your current CPR ability?	Extremely effective	9	5.3	5.3
	Very effective	61	35.7	41.0
	Effective	97	56.7	97.7
	Not so effective	3	1.8	99.4
	Not at all effective	1	0.6	100.0
I would be unsure how to react at work if CPR was needed	Strongly disagree/disagree	137	80.1	80.1
	Neutral	29	17.0	97.1
	Strongly agree/agree	5	2.9	100.0
At work, I consider it my duty to perform CPR in an emergency	Strongly disagree/disagree	8	4.7	4.7
	Neutral	13	7.6	12.3
	Strongly agree/agree	150	87.7	100.0
I would be unsure how to react in public if CPR was needed	Strongly disagree/disagree	124	72.5	72.5
	Neutral	37	21.6	94.2
	Strongly agree/agree	10	5.8	100.0
I would need gloves, face- mask, and other protective items to perform CPR	Strongly disagree/disagree	82	48.0	48.0
	Neutral	45	26.3	74.3
	Strongly agree/agree	44	25.7	100.0
I would prefer not to do mouth-to-mouth during CPR	Strongly disagree/disagree	50	29.2	29.2
	Neutral	44	25.7	55.0
	Strongly agree/agree	77	45.0	100.0

No significant differences were found when questions relating to the use of CPR in work and public settings were analysed by sex, age, years of experience, work setting or work type. The only exception was a significant difference (χ2 (4) = 15.085, *p* = 0.005) in the response relating to consideration of duty to perform CPR in an emergency in a work setting with more podiatrists in private practice than other settings agreeing that it was their duty to perform emergency CPR (private practice 92 %; other workplace settings 68 %).

### Knowledge of CPR principles and practice

Table 4 shows, in descending order, the number (and percentage) of participants who correctly answered each true/false statement on CPR protocols. Most respondents (94 %) reported the correct ratio of compressions to ventilations, not stopping CPR after 15–20 min of resuscitation if the patient had not recovered (92 %), and the correct depth of cardiac compressions (84 %). Most participants also responded correctly on statements relating to the length of breathing check in an unresponsive patient (79 %), the use of automated external defibrillators (AED) on young children (66 %), the first step at a medical emergency (61 %), and seeking help first when alone with a patient needing CPR (58 %). Less than half (49 %) responded correctly to statements on the length of each rescue breath, the AED advising a shock for all cardiac arrests (44 %), the 100–120 min^− 1^ compression rate during CPR (49 %), and reassessing the patient every two minutes to see if they have recovered (20 %).

**Table 4:** Knowledge of current Australian and New Zealand Committee on Resuscitation (ANZCOR) protocols

Statement	True/False	Correct *n %*		Incorrect *n %*	
Correct compressions/ventilations ratio on an adult patient is 30:2 (*n* = 157)	True	148	94.3	9	5.7
Stop CPR if patient not recovered after 15–20 min (*n* = 158)	False	145	91.8	13	8.2
The recommended compression depth for adults during CPR is > 5 cm (*n* = 158)	True	132	83.5	26	16.5
Take no longer than 10 s to check for breathing in an unresponsive patient	True	124	79.0	33	19.3
The AED can be used on infants and children under 8 years of age (*n* = 158)	True	104	65.8	54	34.2
The first step at a medical emergency is to check if the victim is responsive (*n* = 158)	False	97	61.4	61	38.6
If alone with adult patient go for help before starting CPR (*n* = 158)	True	91	57.6	67	42.4
Each rescue breath during CPR should take 1 s (*n* = 158)	True	78	49.4	80	50.6
Compression rate during CPR is 80–100 per minute (*n* = 158)	False	78	49.4	80	50.6
The AED will advise a shock for all victims of cardiac arrest (*n* = 158)	False	69	43.7	89	56.3
Reassess the victim after every 2 min of CPR to check for recovery (*n* = 158)	False	32	20.3	126	79.7

No significant differences were evident in the knowledge of protocols when data were analysed by sex, except for the statement relating to seeking help if alone before starting CPR; more females than males (62 % v 44 %) provided a correct response (χ2 (1) = 4.159, *p* = 0.041). No significant differences were evident in CPR knowledge when data were analysed by age group, work setting, work status, or postgraduate qualification. One significant difference in CPR knowledge was evident in the correlational analysis of length of work experience, with more correct responses among podiatrists with less experience (< 10 years, 60 %; >10 years, 41 %) (χ2 (1) = 5.702, *p* = 0.017).

To measure CPR theoretical knowledge, correct scores for the 11 true/false questions were summated; a score of 11 meant that all responses were correct. The median score of 15 indicated that four questions had been incorrectly completed. Most respondents (76 %) achieved a score of 16 or less, indicating that most questions (seven questions) were correctly answered. No significant differences in total CPR knowledge scores were evident when analysed by age, sex, work setting or status, and years of clinical experience.

## Discussion

This study is the first to investigate the knowledge and perceptions of CPR amongst podiatrists. The overall findings are reflective of prior research with physiotherapists in New Zealand [28], for which 688 physiotherapists completed an online survey investigating their CPR knowledge and qualifications, beliefs about CPR use in an emergency, and perceptions of CPR competency. Participants generally had sound theoretical knowledge, but some significant knowledge gaps did exist among some older practitioners. One in five survey respondents had performed CPR in an emergency, and 81 % had current certification, despite it not being a requirement of registration for physiotherapists in New Zealand. Physiotherapy and podiatry are allied health providers who commonly deliver services in similar primary healthcare settings. Therefore, a comparison with the current findings for New Zealand podiatrists is of interest.

In the current study, 60 % of podiatrists reported having received formal CPR training in the past 12 months and 97 % within the past two years. This finding is expected as, unlike physiotherapy, current CPR certification (within two years) is required for podiatry registration and annual practising certificate in New Zealand. Further, 16 % of podiatrists reported use of CPR in an emergency with a success rate of 50 %. This was similar to the survey of New Zealand physiotherapists in which 19 % reported use of CPR with a 56 % survival rate [28]. Podiatry and physiotherapy are analogous in terms of practice setting and patient demographic, and parallels in the incidence of patients suffering from cardiac arrest during treatment could be expected. However, it is unknown whether participants in the current or previous [28] study performed CPR in the workplace or as bystanders in the community.

Podiatrists were highly confident in their CPR ability, with nearly all respondents (98 %) rating themselves as effective, very effective, or extremely effective. This finding was similar to the New Zealand physiotherapy study in which 92 % of respondents said their CPR ability was at least satisfactory, and 53 % rated their ability as effective or very effective [28]. Most podiatry practitioners (88 %) and physiotherapists (90 %) [28] similarly believed it was their duty to intervene and perform CPR in an emergency in their workplace. This was particularly true for podiatrists in private practice (92 %) compared to other work settings (68 %). By comparison, a recent study of 10,393 healthcare professionals in China found that 74 % were willing to perform bystander CPR on strangers in the community. Those with CPR experience, adequate knowledge and recent training were more likely to perform bystander CPR [32].

Despite a high level of confidence, podiatrists demonstrated varied theoretical knowledge of CPR. The percentage of correct answers for the CPR protocol statements ranged from 20 to 94 %, and percentage scores of less than 50 % were reported for four of the 11 statements. The lowest percentage of correct answers (20 %) related to the statement, “reassess the patient every two minutes to see if they have recovered”, which is false. CPR is not intended to re-start the heart, and interruptions to CPR have been shown to worsen patient outcomes [1]. It is possible that participants who responded incorrectly (believing the statement to be true) may not fully understand the purpose of CPR nor appreciate that providing CPR to a person who does not need it, does not cause harm to the patient. When comparing participant knowledge against demographics (sex, age group, work setting, years of practice and postgraduate qualification), podiatrists with less clinical experience (< 10 years) demonstrated greater knowledge with more correct answers than those with greater clinical experience (> 10 years). This finding may suggest a degree of complacency in more experienced practitioners concerning the need for ongoing training and the assumption that CPR practice guidelines remain unchanged over time.

Similar gaps in CPR knowledge were evident in the study in physiotherapists [28]. Furthermore, a recent study of nurses also found that chest compression psychomotor skill quality was only retained for six months post-training [33], highlighting the need for regular CPR updates. Indeed, the New Zealand Resuscitation Council recommends repeated refresher training for individuals who are not performing resuscitation regularly – stating that individuals should refresh their CPR skills annually [34]. Currently, CPR certification, including AED use and anaphylaxis management, is compulsory for New Zealand podiatrists regardless of practice setting and must be updated biennially [9]. However, in Australia and the United Kingdom (within the National Health Service), annual CPR re-certification is required for podiatrists to maintain an annual practising certificate [35, 36]. The Resuscitation Council UK [37] and Australian Resuscitation Council [1] also recommend that those trained in CPR should refresh their skills at least annually.

There may be some merit in considering a move to annual CPR certification for podiatrists, but this would need to be weighed up against the increased financial implications of course fees and time off work. In addition, a mandated change from biennial to annual CPR re-certification would require the cooperation of the responsible authority (PBNZ) which administers CPD. This change would bring podiatry in line with other health professionals in New Zealand, such as midwives, who require annual CPR re-certification [38]. However, CPR certification is not a registration requirement for nurses, occupational therapists and physiotherapists who are frequently part of the multidisciplinary team involved in podiatry patients’ care. Furthermore, CPR certification is only required for initial registration for doctors, and after that, it is regulated by the employer or the college to which the doctor belongs [39].

## Limitations

The study is not without limitations. The cross-sectional nature of the survey allows for the determination of associations only, rather than causality. The use of self-reported data on CPR may have introduced bias that may not reflect actual behaviours [40]. It is unknown whether participants performed CPR in the workplace or as a bystander in the community. CPR ‘success’ was not defined in the survey. Therefore, the question, “If yes, was it [CPR] successful?”, was open to participant interpretation. Knowledge of CPR protocols assessed in written form is not a true reflection of competence. Future research evaluating actual competency, through a practical assessment on a manikin, would be valuable and inform a potential move to annual CPR re-certification.

## Conclusions

Podiatrists have a duty of care to assist patients in a medical emergency, and current CPR certification is a requirement for registration in New Zealand. This study is the first to investigate podiatrists’ CPR knowledge and perceptions using a web-based survey. Podiatrists were found to have high levels of CPR confidence but demonstrated gaps in CPR knowledge. Similar findings in New Zealand registered physiotherapists prompted a call for mandatory CPR certification as an ongoing continuing medical education requirement. Currently, New Zealand registered podiatrists require biennial CPR re-certification. However, resuscitation authorities in New Zealand and overseas recommend an annual update of CPR skills. As New Zealand’s population ages, podiatrists will increasingly encounter medical emergencies requiring CPR intervention, and preparedness to administer CPR competently and confidently is critical to meeting their professional obligations. Based on the findings of this study, and in line with Australia and the UK, the authors recommend a change from biennial to annual CPR re-certification for podiatrists in New Zealand.

## Supplementary Information

**Additional file 1: **Survey questions

## Data Availability

The datasets generated or analysed during the current study are available from the corresponding authors on reasonable request.

## Abbreviations

CPR: Cardiopulmonary resuscitation
AED: Automated external defibrillator
ANZCOR: Australian and New Zealand Committee on Resuscitation
PBNZ: Podiatrists Board of New Zealand
