# Supplementary material for: Knowledge and perceptions of cardiopulmonary resuscitation amongst New Zealand podiatrists: a web-based survey
Source: J Foot Ankle Res. 2021 May 14;14:40. doi: 10.1186/s13047-021-00481-9 (PMC8120252; doi:10.1186/s13047-021-00481-9)
Supplement: Supplementary file 1 — Additional file 1: Survey questions [file 13047_2021_481_MOESM1_ESM.pdf]

Survey

Introduction

Hello and welcome to this survey exploring the knowledge, attitude and practice of cardiopulmonary resuscitation (CPR) among New Zealand podiatrists. The survey contains questions about your current knowledge of CPR and your attitude about how and when you would use CPR.

The survey will take approximately 5-10 minutes to complete. Please make sure you answer all the questions.

There are three sections: demographics, CPR practice/attitude and CPR knowledge.

Please read each question carefully and click the appropriate box (tick one box only per question).

1. Sex?

- ☐ Male
- ☐ Female

2. Age?

- ☐ 20-29
- ☐ 30-39
- ☐ 40-49
- ☐ 50-59
- ☐ 60+

3. Years of podiatry experience?

- ☐ 0-5
- ☐ 6-10
- ☐ 11-15
- ☐ 16+

4. Postgraduate qualification?

- ☐ Yes
- ☐ No

5. Current employment status?

- ☐ Employed
- ☐ Self-employed
- ☐ Employer
- ☐ Other

6. Main work setting

- ☐ Private practice
- ☐ Public hospital/clinic
- ☐ Community
- ☐ University
- ☐ Other

CPR practice/attitude

Please read each question carefully and click the appropriate box (tick one box only per question).

7. When was your last formal CPR resuscitation training?

- ☐ <3 months
- ☐ 3-12 months
- ☐ 1-2 years
- ☐ 2+ years

8. Did your last CPR training include automated external defibrillator (AED) training?

- ☐ Yes
- ☐ No

9. Did your last CPR training include anaphylaxsis management?

- ☐ Yes
- ☐ No

10. Did your last CPR training involve practical face-to-face teaching?

- ☐ Yes
- ☐ No

11. In relation to your main place of work, do you know where your nearest AED is located?

- ☐ Yes
- ☐ No

12. Have your ever used CPR in an emergency?

- ☐ Yes
- ☐ No

13. If you have ever used CPR (or the last time you use it), did the patient survive?

- ☐ Yes
- ☐ No
- ☐ Don't know
- ☐ Not applicable

14. What do you believe the survival rate of patients is, for out of hospital cardiac arrest?

- ☐ 0-25%
- ☐ 26-50
- ☐ 51-75
- ☐ 76-100%

15. How would you rate your current CPR ability?

- ☐ Not at all effective
- ☐ Not so effective
- ☐ Somewhat effective
- ☐ Very effective
- ☐ Extremely effective

16. I would feel unsure of how to react at work, if I was presented with an emergency situation requiring CPR:

- ☐ Strongly disagree
- ☐ Disagree
- ☐ Neither agree nor disagree
- ☐ Agree
- ☐ Strongly agree

17. At work, I would consider it my duty to intervene in an emergency situation and perform CPR:

- ☐ Strongly disagree
- ☐ Disagree
- ☐ Neither agree nor disagree
- ☐ Agree
- ☐ Strongly agree

18. I feel unsure of how to react if I was presented with an emergency situation in public (outside of work) requiring CPR:

- ☐ Strongly disagree
- ☐ Disagree
- ☐ Neither agree nor disagree
- ☐ Agree
- ☐ Strongly agree

19. I would need gloves, face mask and other items relevant to self-protection to perform CPR:

- ☐ Strongly disagree
- ☐ Disagree
- ☐ Neither agree nor disagree
- ☐ Agree
- ☐ Strongly agree

20. I would prefer not to perform mouth to mouth ventilation during CPR:

- ☐ Strongly disagree
- ☐ Disagree
- ☐ Neither agree nor disagree
- ☐ Agree
- ☐ Strongly agree

CPR knowledge

Please read each question carefully and click the appropriate box.

21. The compression rate during CPR is 80-100 per minute

☐ True ☐ False

22. Each rescue breath during CPR should be given over a 1 second period

☐ True ☐ False

23. If alone with an adult patient, go for help before starting CPR

☐ True ☐ False

24. Stop CPR if the patient has not recovered after 15-20 minutes of resuscitation

☐ True ☐ False

25. The AED will advise a shock for all cardiac arrest patients

☐ True ☐ False

26. Take no longer than 10 seconds to check for breathing when assessing an unresponsive patient

☐ True ☐ False

27. Reassess the patient after every two minutes of CPR to see if they have recovered

☐ True ☐ False

28. The AED can be used on infants and children under 8 years of age

☐ True ☐ False

29. The first step at the scene of a medical emergency is to check if the patient is responsive

☐ True ☐ False

30. The correct ratio of compression to ventilations on an adult patient during CPR is 30:2

☐ True ☐ False

31. The recommended compression depth for adults during CPR is >5cm

☐ True ☐ False
